# Optimal Scheduling and Fair Service Policy for STDMA in Underwater Networks with Acoustic Communications

**DOI:** 10.3390/s18020612

**Published:** 2018-02-17

**Authors:** Miguel-Ángel Luque-Nieto, José-Miguel Moreno-Roldán, Pablo Otero, Javier Poncela

**Affiliations:** Department of Ingeniería de Comunicaciones, University of Málaga, Málaga 29010, Spain; jmmroldan@uma.es (J.-M.M.-R.); pablo.otero@uma.es (P.O.); jponcela@uma.es (J.P.)

**Keywords:** optimal scheduling, time-division multiple access, Gini index, string network, underwater sensor networks

## Abstract

In this work, a multi-hop string network with a single sink node is analyzed. A periodic optimal scheduling for TDMA operation that considers the characteristic long propagation delay of the underwater acoustic channel is presented. This planning of transmissions is obtained with the help of a new geometrical method based on a 2D lattice in the space-time domain. In order to evaluate the performance of this optimal scheduling, two service policies have been compared: FIFO and Round-Robin. Simulation results, including achievable throughput, packet delay, and queue length, are shown. The network fairness has also been quantified with the Gini index.

## 1. Introduction

Underwater sensor networks (UWSNs) have great potential in many areas, mainly in environmental monitoring, with applications in the fields of oceanography, defense and security, and fisheries, which include pollution control, the gathering of scientific data, or intruder’s surveillance. Image transmission from remote sites is the most envisaged capability of UWSNs [1,2,3]. One important case is monitoring the behavior of river-fed sediment plumes in estuaries and deltas [4], because of their influence on water quality and the environment.

UWSNs are long-overdue to reduce the cost of traditional monitoring methods, like campaigns of CTD (Conductivity-Temperature-Depth) and turbidity measurements on board ships. These measurement campaigns are also affected by bad weather, e.g., storms, which are frequently the reason for the campaigns. Furthermore, the data gathered and sent by the network can be available near real time in an on land data center for later processing.

Nowadays, using optical wireless communications for the underwater medium is only possible up to a few meters [5], and even less if radiofrequency waves are used instead, while acoustic waves can reach distances in the range of kilometers. Therefore, the communication channel chosen in this work is the underwater acoustic channel (UAC), which has several important limitations: the low propagation speed of the acoustic waves, the limited available bandwidth, the high absorption, and the frequency-selective fading. The former causes underwater communications to suffer from a high latency, while the others only allow for transmission with a limited signaling rate. Special care must be taken in time scheduling to achieve a reasonable throughput in the network operation. In this paper, we cope with the latency so that it will not play against throughput.

The first step to achieve a high throughput must be to employ a time-efficient MAC layer. In this work, the Time-Division Multiple Access (TDMA) operation has been adopted, since it is the most time-efficient technique [6]. Nevertheless, TDMA requires good scheduling to organize the node transmissions. We exploit the large propagation delay to choose the best scheduling, as we will see later in Section 2.

Many TDMA techniques have already been presented and assessed in the literature when concerning the MAC layer in UWSNs [7]. A recent paper that also provides an exhaustive survey of the different TDMA variants in this type of networks is [8]. For example, the protocol named the Acoustic Communication network for Monitoring of Environment (ACMENet) [9], divides sensor nodes into two types: master and slave nodes, similar to protocol SBMAC [10]. In these protocols, propagation delays are measured and the resulting values are used to avoid packet collisions. Although slave nodes in the ACMENet protocol have a simple design, the master node is complex and can be a problem when the network size grows. In [11], protocol ST-MAC is designed to overcome the spatial-temporal uncertainty in the TDMA-based MAC scheduling, improving the throughput by means of resolving a conflict graph. 

Spatial TDMA (STDMA) was proposed as early as 1985 [12]. The idea is that two hop links of the network that are far enough apart, i.e., do not interfere each other, can be operating simultaneously. Scheduling is of paramount importance in STDMA networks. Luque-Nieto, et al. [4] present optimal STDMA scheduling for linear networks where the sink node collects a single packet from every node in one frame. The problem of finding the shortest frame is addressed as a bin packing problem. However, no propagation delay was considered. The long propagation delay of the acoustic waves allows the nodes to overlap their transmissions in time without collisions. The idea of exploiting the time overlapping of travelling waves to increase the throughput in TDMA networks has been already proposed [6,13,14]. It has been successfully applied to several topologies, like a grid mesh of sensors [15] or in a linear network [16], by means of dynamic linear programming. Nevertheless, these methods, based on a sequential decision problem slot by slot, are far from easy to implement in networks with a medium/large number of nodes. A new function to obtain a figure of merit of the vector of the states, denoted reward in [13], must be optimized by iteration for each network size. In these networks, the main drawback is the need to ascertain an efficient and simple algorithm to determine the optimal schedule with low complexity. In the case of a regular spaced linear network, another approach to find an optimal schedule is to analyze the constraints to avoid collisions within the dual space-time domain (location of nodes and time slots) in a geometric 2D lattice chart [17]. We now show in this work that it is possible to use the 2D space-time lattice to find the best possible schedule (optimal) for a linear network.

A key issue in UWSN is the energy consumed by the nodes. In [18], a procedure to avoid retransmissions is pointed out by means of transmitting duplicate data through different paths of the network (routing). Nevertheless, this topic is not the focus of the present work. Another key point in UWSNs is the physical topology. We consider a static monitoring network with fixed nodes anchored to the seabed, which is a realistic assumption for a monitoring or surveillance UWSN [16]. According to the number of nodes, and the area to cover for sensing, we can find the formation ranging from simple isolated linear networks (called string or chain networks) up to clusters of subnetworks linked through special nodes acting as master nodes. In this work, we have chosen a simple case, the multi-hop string network, but under the worst operation conditions: the sink node is located at one end of the string. This fact may create a bottleneck that needs to be overcome. As a consequence, multi-hop TDMA network scheduling has two components: 1) A time schedule to assign transmission slots to nodes and 2) a packet service policy to determine the origin of the packet to be transmitted in a particular time slot. In this paper, scheduling is proposed so that the network performance is optimal in terms of throughput and fairness. In order to obtain the packet end-to-end delay, two services policies are compared: FIFO and Round-Robin.

Probabilistic wireless networks can be modeled and analyzed using process calculus [19,20] describes a compositional theory for networks with static topology assuming that communication between nodes is reliable, and applies the theory to routing protocols. Merro, et al. [21] defines a well-formed network as a network which is node-unique, connected, exposure-consistent, and transmission-consistent. Bugliesi, et al. [22] describes a framework for the analysis of mobile ad-hoc networks (MANET) and proposes the evaluation of communication interference based on preorders; this work also models the concept of a scheduler in wireless networks. The analysis is carried out for well-formed networks in which active receivers are in the range of exactly one transmitter and transmitters sense the channel beforehand. A similar probabilistic approach is used in [23] to address connectivity and energy consumption in mobile wireless sensor networks. 

Networks like the one analyzed in this work can be modelled as an open Jackson network using queueing theory. Each node is considered a service station that receives as input the packets from the previous node plus its own generated packets [24]. In our network, however, we can simplify those more general approaches as nodes generate traffic in a deterministic way, and we use spatial TDMA multiplexing [12] with fixed scheduling. This means that the arrival times at each node are deterministic, as well as the instants of the generation of packets in each node. Thus, we can see our network as a special case of the previous analysis frameworks. These assumptions model a deployable monitoring network where information is sent periodically towards a central facility. Our aim is to identify the schedule and policy that maximize the amount of information that can be received under a fairness constraint.

The rest of the paper is organized as follows. Section 2 introduces the network model including the operation of the nodes and the collision constraints involved. Then, time scheduling and some related points are addressed, especially the optimal scheduling. A new graphic method to find the optimal scheduling is presented. Section 3 analyses the network performance for the two service policies considered: FIFO and Round-Robin. Firstly, numerical results for throughput are presented. Secondly, end-to-end packet delay is measured and improved by establishing specific initial conditions. Thirdly, the mean queue length of the nodes is calculated. Finally, concerning the fairness behavior, a discussion about the delivered packet distribution to the Gateway node is presented.

## 2. Network Model and Operation 

**Figure 1:**
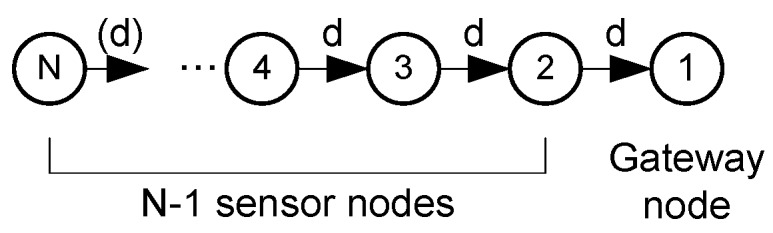
String network with a set of N−1 equidistant (distance *d*) sensor nodes and a single Gateway node (node 1) at the extreme.

The string network under study is shown in Figure 1. There are two kinds of nodes: the sensor nodes (numbered from 2 to N) and a single sink node (the Gateway, numbered 1) located at the network edge. The seabed is neither flat nor smooth. Therefore, nodes may not be equally spaced. Nevertheless, the analysis will begin considering that the nodes are equally spaced in terms of the distance *d* between each of them and eventually, in Section 2.2.3, we will consider the general case of unequally spaced nodes. 

The sensor nodes provide fixed size data packets (e.g., with environmental measurements) and the Gateway node collects all the packets to forward them to a data center normally located on the water surface or on land, for later processing. The nodes will use a unicast service, so the packets of a node will be routed to the next node toward the Gateway (path in Figure 1). This multi-hop routing technique has the advantage of saving energy, an important issue in UWSNs.

**Figure 2:**
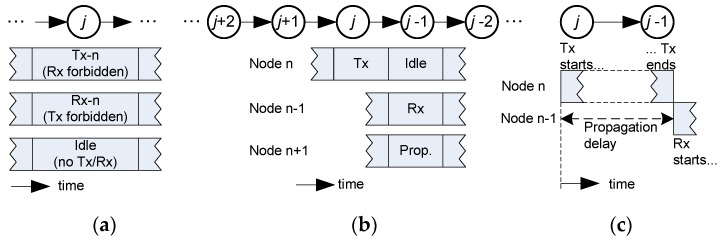
Network operation: (**a**) half-duplex operation means a sensor node can be in three states: transmission, reception, or idle; (**b**) omnidirectional propagation (with one hop power control): when node *j* transmits, the propagated wave reaches both nodes *j* + 1 and *j* − 1, but only node *j* − 1 receives the data (following the routing path); (**c**) the transmission time equals the propagation delay.

Concerning the communication technique employed, the following considerations are assumed: (i) one-way operation; (ii) non-directive transducers (projectors and hydrophones) with a power range adjusted to a single-hop distance; and (iii) a transmission time, the so-called time slot, equal to the propagation delay to reach the neighbor node. These starting conditions are pointed out in Figure 2. Because of (i), a sensor node can be in one of three states: transmission, reception, or idle. Condition (ii) allows the acoustic wave to travel in all directions from a transmitting node, reaching every neighbor node in its transmission range, and causing interference. Due to the power control applied to the acoustic modem, it is possible to establish a hop-by-hop relayed operation with simultaneous transmissions from nodes far enough from each other, because the acoustic power received from farther than two hops will be negligible. Power control is currently a mature technology, widely used in wireless systems. Nodes transmit the power strictly needed to reach their respective destination, which substantiates the assumption of negligible interference at the second hop. Condition (iii) ensures that the time slot available to transmit is full of data, and will enhance the throughput obtained.

### 2.1. Collision Constraints

**Figure 3:**
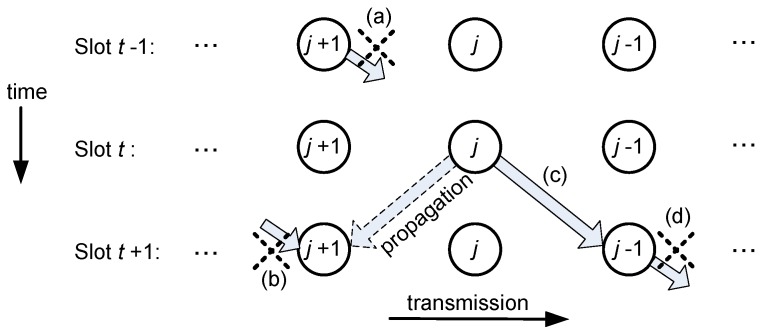
Collision-free constraints when node n transmits in time slot *k*. (**a**) In the previous slot t−1, node *j* + 1 cannot transmit because node *j* will be busy transmitting in time slot t and will not receive the data. (**b**) Node *j* + 1 is not allowed to receive in time slot *t* + 1 to avoid the collision between the wave coming from node j and the expected wave from node *j* + 2. (**c**) Node *j −* 1 must be receiving the wave from node *j* in time slot *t*+1, so that it cannot transmit in that time slot (**d**).

Let us consider that node *j* has to transmit in time slot *t*. If the transmission range is one hop (distance *d* in Figure 1), there are three constraints to avoid interference: (i) node j+1 cannot transmit in the previous slot t−1; (ii) the destination node j−1 cannot transmit in slot t+1, when it expects to receive the data from node *j*; and (iii) nodes j and j+2 cannot transmit simultaneously. The reasons for these constraints are discussed in Figure 3 with the help of a graphic sketch. Besides that, since the network has a finite size, there are two obvious rules for nodes at both ends: the Gateway (node 1) never transmits to another node in the network, and node N never receives data from another node. These conditions will be considered in Section 2.2.2 to find valid schedules.

### 2.2. Time Schedule

Time scheduling in a TDMA network concerns how to assign time slots to nodes for transmission. If the assignment is periodic, the shortest complete set of slots without repetition is the so-called frame. In a TDMA network, a schedule is a periodic sequence of time slots (frame), where every time slot can be assigned to a set of nodes. Due to the constraints mentioned in the previous Section, transmission is not possible during all the time slots in a frame. A first goal in scheduling is to make as many transmissions as possible within a frame to increase the throughput in the network, while keeping the frame as short as possible, avoiding the collisions already mentioned.

#### 2.2.1. Definitions

A schedule can be represented by a matrix Q [19], where element $q_{t,j}\;$denotes the state of node *j* during time slot t: $q_{t,j}=\ell$ when node j will be planned to transmit to node ℓ, and $q_{t,j}=-\ell$ when node j will be planned to receive from node ℓ. Finally, $q_{t,j}=0\;$means node j is in an idle state.

A schedule has a period *T* if it repeats every *T* time slots: $q_{t,j}=q_{t+T,j\;\;}\forall t,j$. In essence, TDMA has a periodic operation, so our interest will be focused on periodic schedules. Period *T* is the frame length. We denote by $Q^{\left(T\right)}$ the matrix that represents the schedule in a period.

A perfect schedule is a matrix $Q^{\left(T\right)}$ with no zero entries (no node has an idle state in the frame). A schedule is called optimal in case of providing maximum throughput. In [19], it is shown that every network has an optimal schedule that is periodic, so the periodicity is an important feature to search for the optimal schedule. Since it is impossible to find a perfect schedule for a linear array of N>2 nodes [19], our goal is to find an optimal periodic schedule containing the largest number of transmissions or, which is the same, the smallest number of idle states.

The average throughput S of a periodic schedule $Q^{\left(T\right)}$ can be calculated as the number of either transmissions or receptions in one frame divided by the frame length [19,25]:

(1) $$S\;=\;\frac{1}{T}\begin{array}{c} \sum_{t,j}^{}\begin{array}{l} 1_{\left(q_{t,j}<0\right)} \end{array} \end{array},$$

where $1_{\left(A\right)}$ is the indicator function with value 1 if the logical expression A is true and 0 otherwise.

The constraints shown in Figure 3 can be mathematically expressed for any discrete time *t* as follows:

(2) $$q_{t,j}=\;–\;\ell \;\;\;\overset{\;}{\Rightarrow }\;\;\ell =\;j+1\;\;\;\;\;\;\;\;\;\mathrm{with}\;\;j\in \left\{2,\;3,\;\ldots ,\;N–1\right\}\;,$$

(3) $$q_{t,j}\;=\;j\;–\;1\;\;\overset{\;}{\Leftrightarrow }\;q_{t+1,j-1}=\;–\;j\;\;\;\;\;\;\;\mathrm{with}\;\;j\in \left\{2,\;3,\;\ldots ,\;N–1\right\}\;,$$

(4) $$q_{t,j}\;=\;j\;–\;1\;\;\overset{\;}{\Rightarrow }\;q_{t,j-2},\;q_{t,j+2}\leq 0\;\;\;\;\;\;\;\mathrm{with}\;\;j\in \left\{3,\;\ldots ,\;N–2\right\}\;,$$

(5) $$q_{t,1}\;\in \;\left\{0,\;–2\right\},$$

(6) $$q_{t,N}\;\in \;\left\{0,\;N–1\right\}.$$

Equations (2) and (3) mean that a node can only transmit to its down-stream neighbor, Equation (4) states that nodes j and j+2 cannot transmit simultaneously, and Equations (5) and (6) set the boundary conditions for a finite network: Equation (5) means that the Gateway (node 1) either collects packets from node 2 or remains idle, and Equation (6) means that the upper-stream node N never receives packets from other nodes. It is important to note that positive values represent nodes in a transmission state, negative values represent nodes in a reception state, and zero means idle. Since a transmission is always associated with a reception, there will be as many positive valued elements as negative ones. 

#### 2.2.2. Space-Time Analysis

**Figure 4:**
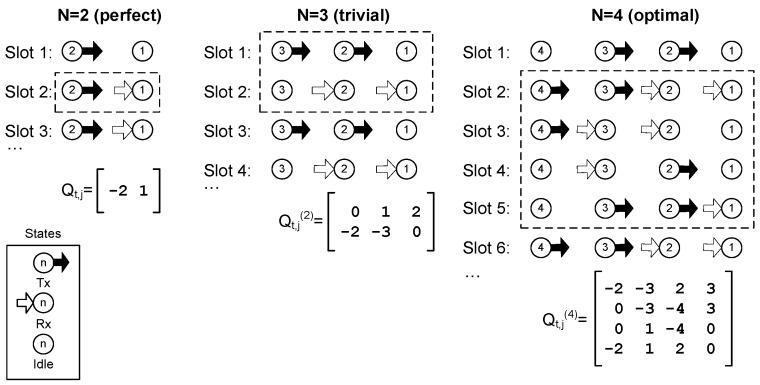
Examples of efficient schedules for different network sizes (N). Matrix $Q^{\left(T\right)}$ is provided for each case, and the first frame is highlighted in a dashed line. The perfect schedule is only possible for N=2, with period T=1. If N=3, the solution is trivial, with period T=2. For N=4, the optimal solution is shown (T=4 ).

In order to find a simple algorithm that in turn finds the optimal schedule of the string network, we start analysing a small network. Later on, in Section 3.1, the limitation on the packet generation rate of every node for queue stability will be established. The optimal schedules found for network sizes N= 2, 3, and 4 nodes are shown in Figure 4. The procedure to obtain the optimal schedule consists of applying Equations (2)–(6) to the nodes, trying to minimize the number of idle states, and looking for a periodic operation. It can be noted that for N= 4, slots 6 and 2 are equal, and the same will happen with 7-3, 8-4, and so on. Therefore, for *N* = 4, the frame length is *T* = 4. In this schedule, there are only four nodes in an idle state during the whole frame, achieving the busiest operation possible for this network topology.

The optimal schedule found for N=4 (see Figure 4) has a period of *T* = 4. It happens that the optimal frame length is also *T* = 4 for *N* > 4. The method to obtain $Q^{\left(T\right)}$ can be extended to any network size, because of the regularity of the network structure (hop distance and conditions of the edge nodes). First, we must define a set of four column vectors$\;v_{j}^{\left(i\right)}$ for each node *j* of the network, i∈{0,1,2,3}:

(7) $$v_{j}^{\left(0\right)}={\left(\;\left[\begin{array}{cccc} j-1 & j-1 & -\left(j+1\right) & -\left(j+1\right) \end{array}\;\right]\;\right)}^{\prime }\;,$$

(8) $$v_{j}^{\left(1\right)}={\left(\;\left[\begin{array}{cccc} -\left(j+1\right) & j-1 & j-1 & -\left(j+1\right) \end{array}\;\right]\;\right)}^{\prime }\;,$$

(9) $$v_{j}^{\left(2\right)}={\left(\;\left[\begin{array}{cccc} -\left(j+1\right) & -\left(j+1\right) & j-1 & j-1 \end{array}\;\right]\;\right)}^{\prime },$$

(10) $$v_{j}^{\left(3\right)}={\left(\;\left[\begin{array}{cccc} j-1 & -\left(j+1\right) & -\left(j+1\right) & j-1 \end{array}\;\right]\;\right)}^{\prime }\;,$$

where the superscript ‘ stands for transpose. Note that, in this case, $v_{j}^{\left(i\right)}$ represents column vectors, i.e., j is the column index of matrix $Q^{\left(T\right)}$. It is easy to see that two elements of each vector Equations (7)–(10) will always be negative. These elements represent nodes in a reception state.

**Figure 5:**
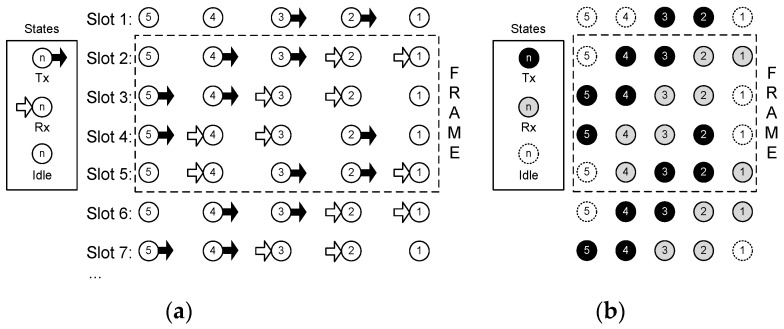
(**a**) State diagram for the optimal schedule in a string network with N=5. An equivalent but more convenient representation to apply a geometrical method is shown in (**b**).

Finally, the optimal schedule for a string network of N nodes, which we denote by $Q_{opt,4\times N}^{\left(4\right)}$, is obtained using vectors Equations (7)–(10) in the columns of the *Q* matrix corresponding to the node number in the subscript *j* of each vector$\;v_{j}^{\left(i\right)}$, and the superscript *i = j* modulo 4. That is, the optimal schedule is:

(11) $$Q_{opt,\;\;4\times N}^{\left(4\right)}=\left[\begin{array}{cccccccc} v_{1}^{\left(1\right)} & v_{2}^{\left(2\right)} & v_{3}^{\left(3\right)} & v_{4}^{\left(0\right)} & v_{5}^{\left(1\right)} & v_{6}^{\left(2\right)} & \cdots & v_{N}^{\left(N\;\mathrm{mod}\;4\right)} \end{array}\right],$$

where, in the last vector $v_{N}^{\left(N\;\mathrm{mod}\;4\right)}$, the negative elements must be replaced by zero because node *N* never receives from another node and, instead, it will remain in the idle state. For example, Figure 5 shows the optimal schedule for a string network of size N=5, which is given by:

(12) [v1(1)v2(2)v3(3)v4(0)v5(1)]=[−2−323−60−3−43401−4−54−212−5−6] ⇒ Qopt,4×5(4)=[−2−32300−3−43401−4−54−212−50].

**Figure 6:**
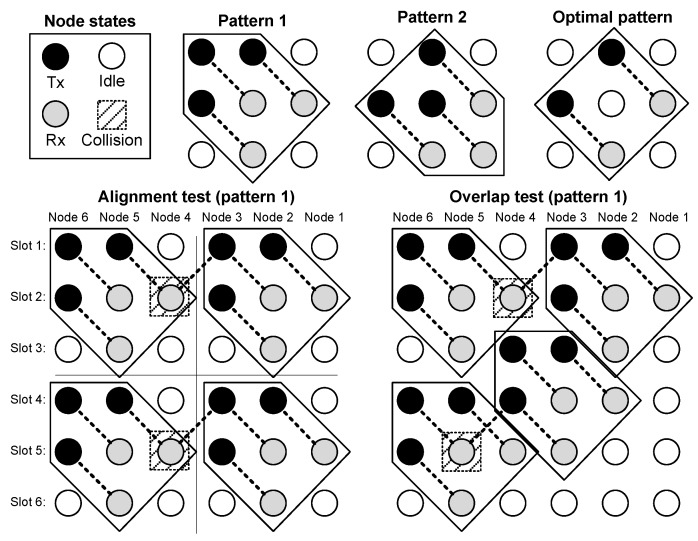
Example search of the optimal schedule for a string network with six nodes. In the upper-left corner, there is the legend. To its right, the three patterns considered (1, 2, and optimal) with a size of 3×3 (time slots × nodes). At the bottom, test results for pattern 1 are shown: alignment (**left**) and overlap (**right**). Both tests indicate collisions in the 2D space-time lattice, so pattern 1 is not a valid pattern.

A geometric interpretation can be given to find and to prove this optimal schedule. Let us imagine a 2D space-time lattice, where the node number is in the x-axis and the time slot number is in the y-axis. The problem can be formulated as to set the state (Tx/Rx/Idle) of every node in that 2D space-time lattice, with two constraints: minimize the number of idle states and be periodic in time. We call the pattern the set of states that is repeated in space and time. A final consideration concerns the size of the pattern: the minimum size is 3×3, because 2×2 is trivial, and does not lead to a valid solution when it is repeated within the 2D lattice. The proposed procedure to find the best scheduling includes three steps. In the first step, called pattern selection, a reduced state pattern (time slots × nodes with Tx/Rx assigned states), which fulfils Equations (2)–(4), is found, so that it includes as many Tx/Rx states as possible. Once the pattern is found, in the second step, called alignment, the pattern is repeated in the 2D space-time lattice. If collisions arise, the pattern is discarded. When the alignment provides a collision-free schedule, in the third step, called overlap, the Tx/Rx part of a pattern is moved to fill as many idle states as possible and then, again, a check for collisions is carried out, as shown in the lower right corner of Figure 6. In the steps of alignment and overlap, the found schedule must fulfill conditions Equations (2)–(6). Figure 6 shows examples of three candidate 3×3 patterns, which fulfill constraints Equations (2)–(4), but only one of them passes both alignment and overlap tests without collisions. When repeated by overlapping in the whole 2D lattice, this candidate pattern turns into the optimal schedule presented in Equation (11). For the sake of brevity, the alignment and overlap tests have only been included in Figure 6 for pattern 1, but the procedure is similar for the other two patterns (2 and the optimal case). 

#### 2.2.3. Unequally Spaced Nodes

Let us consider now the general case of unequally spaced nodes. To make sure that there will be no interference, in this scenario, the transmission time, which is given by the packet size, cannot be equal to the time slot. To avoid interference, two constraints must be met. First, the time slot must be set so that the longest propagation time is considered; this way, all transmissions will reach their destinations before the end of the second time slot after their start. Second, the packet size must be adjusted to the shortest propagation time; this way, no transmission will reach its destination node before the end of any other possible interference in that node. 

**Figure 7:**
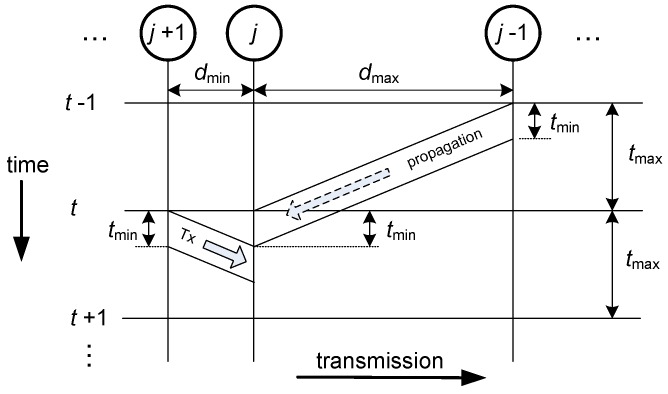
To illustrate the general case of unequally spaced nodes in the event of adjacent shortest and longest links.

These ideas are depicted in Figure 7, where the worst case of the shortest and longest links being adjacent is shown. We denote $t_{\min}$ to be the propagation time of the shortest link, and $t_{\max}$ the propagation time of the longest link. The link distances are, respectively, $d_{\min}$ and $d_{\max}$. In this general case, scheduling is obtained using the proposed method. The throughput will decrease by a factor of $\tau =t_{\min}/t_{\max}=d_{\min}/d_{\max}$ compared to the results for the equidistant network.

## 3. Results: Network Performance 

The performance of the network will be evaluated in four aspects: throughput, end-to-end delay, queue length, and fairness. Closed-form expressions are given for throughput. In case of packet end-to-end delay, we present simulation results. Furthermore, by evaluating different initial conditions, we show that it is possible to reduce the average delay in the network; numerical results are given to prove the statement. The lengths of the queues are determined by simulations. Finally, we have calculated the Gini index to assess the fairness behavior of the network. Simulation results of the Gini index for both throughput and packet delay are shown.

### 3.1. Throughput 

The number of transmissions or receptions in a frame is the number of positive or negative elements of Equation (11). With the proposed method, Equation (11) can be easily built for any N and realize that this number is 2(N−1). A more rigorous explanation is based on counting the negative valued elements (receptions) in Equation (11). Since there are two negative elements in each Equations (7)–(10), and the right end column of the matrix has no negative values, in a string network with N nodes, the optimal scheduling in Equation (11) will have 2(N−1) transmissions/receptions in the frame. Since the period is four time slots, the theoretical average throughput (1) is given by:

(13) $$S=\frac{2\;\left(N-1\right)}{4}=\frac{N-1}{2}\;.$$

The throughput in Equation (13) is close to the throughput of the perfect schedule, which has a value of  N/2 , impossible to obtain in a linear network, as discussed above. The difference is 100/N per cent: the larger N, the smaller the relative difference between the throughputs of both schedules. Obviously, since a node cannot transmit and receive simultaneously, the upper bound of the throughput is $S_{max}=1/2\;.$ We denote $\lambda_{j}$ to be the packet generation rate of node *j* and consider equal offered traffic by all sensor nodes. In order to achieve this upper bound, the packet generation rate of the *j* node, $\lambda_{j}$, can be calculated as:

(14) $$\left(N-1\right)\cdot \lambda_{j}=\frac{1}{2}\;\;\;\overset{}{\Rightarrow }\;\;\lambda_{j}=\frac{1}{2\left(N-1\right)}\;.$$

**Figure 8:**
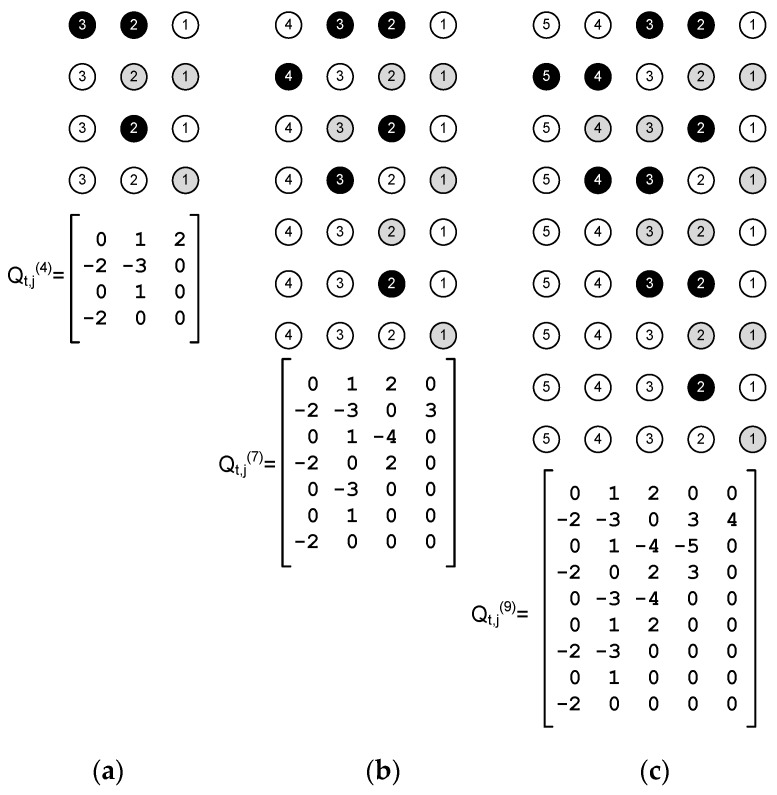
Simple fair schedule (one frame) examples for different string network sizes (N): (**a**) N  = 3 (T  = 4), (**b**) N  = 4 (T  = 7), and (**c**) N  = 5 (T  = 9). The Q matrix is shown for each case. For N=2, the solution is trivial with period T=1. The three states for the nodes are represented keeping the convention used in Figure 5 and Figure 6.

For comparison purposes, we can consider a simple fair schedule where every sensor node only transmits one self-generated packet to the Gateway in a frame (Figure 8). The frame length of this scheduling is given by:

(15) $$T=\{\;\;\;\begin{array}{ll} 7+5\;\frac{N-4}{2} & \mathrm{if}\;N\;\mathrm{even},\\ 4+5\;\frac{N-3}{2} & \mathrm{if}\;N\;\mathrm{odd}. \end{array}$$

Equation (15) is obtained by inspection of the graphics shown in Figure 8, up to N=7. Due to the regularity of the network structure, it is also true for larger networks. A packet generated in node *j* will be relayed j−1 times to reach the Gateway. Therefore, the total number of transmissions in one frame is:

(16) $$\sum_{j=2}^{N}j-1=\frac{N\left(N-1\right)}{2}.$$

The average throughput is the number of transmissions in a frame divided by the frame length. Using Equations (15) and (16) we obtain:

(17) $$S=\frac{\frac{N\left(N-1\right)}{2}}{T}=\{\;\;\;\begin{array}{ll} \frac{N\left(N-1\right)}{5N-6} & \mathrm{if}\;N\;\mathrm{even},\\ \frac{N\left(N-1\right)}{5N-7} & \mathrm{if}\;N\;\mathrm{odd}. \end{array}$$

**Figure 9:**
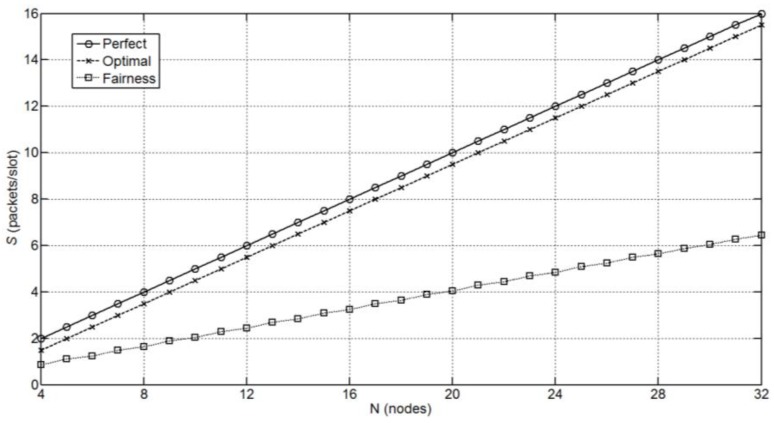
Throughput obtained by the optimal (cross marker) and simple fair (square marker) schedules in a N-nodes string network. In order to compare both with the ideal limit, the throughput for a perfect schedule (circle marker) is also shown.

Figure 9 compares the throughput results of both optimal and simple fair schedules and the perfect schedule, showing that the optimal is the non-perfect schedule with the highest average throughput in the network. The reason not to achieve the throughput of the perfect schedule lies in the border effect: the last node never receives data from another node.

### 3.2. Delay 

One of the most important parameters of the performance of a multi-hop network is the delay of the packets delivered by the nodes to the Gateway, which should be as low as possible. In order to compute the delay, the network has been simulated with a Matlab©-based proprietary discrete events simulator. Two measures have been considered: the maximum and the average end-to-end delay$\;\bar{D}$, defined by:

(18) $$\bar{D}=\frac{1}{P_{tot}}\left(\sum_{j=2}^{N}\sum_{i=1}^{p_{j}}D_{j,i}\right)\;,$$

with $P_{tot}$ being the total number of packets delivered, $p_{j}$ the amount of packets delivered from node *j*, and $D_{j,i}$ the end-to-end delay of the i-th packet from node j. 

**Figure 10:**
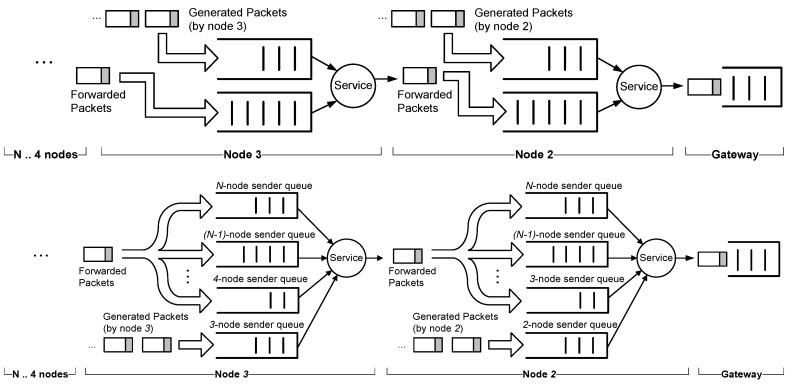
Scheme for two service policies in a string network: FIFO (above) and Round-Robin (below).

In a transmission state, the service policy of the node has to choose which packet to transmit: its own generated packet or a packet from another node (relayed packets). As seen in Figure 10, several strategies for the service can be implemented independently of the scheduling scheme adopted. In order to measure the packet delay, we have considered two strategies: FIFO and Round-Robin. In the case of FIFO policy, we need to include two queues, one for the packets received from the up-stream nodes and another one for the self-generated packets. For the sake of clarity, we will denote the former to be the queue and the latter to be the buffer. For the buffer, we will assume the packet rate in Equation (14).

In the case of Round-Robin policy, node *j* has N−j  different queues that store packets coming from the up-stream nodes (nodes j+1 to N) and the buffer for its own generated packets. In every transmission slot, a different queue is selected by rotation to send a packet. If the selected queue is empty, the next queue in the sequence is chosen. If none of them have packets, it will be the turn of node *j*, which will send a packet from its buffer. 

Both policies, FIFO and Round-Robin, are examples of polling systems. Systems with several queues attended by a single server based on a pre-scheduled rule belong to the family of vacation queues with a gated operation and a time limited service (one single time slot) [26,27]. However, since the transmissions are scheduled, the arrivals process is deterministic and can be studied without resorting to queueing theory. Both delays are shown in Table 1 for a set of network sizes varying from N=4 to N=100. It can be seen that the delays are almost equal in both cases. The FIFO service policy is then preferable for large *N* because of an easier implementation since there are only two queues per node. Last but not least, the initial conditions different from perfect synchronism, i.e., all the nodes start the counter to transmit from their own buffer simultaneously, can slightly affect the delay shown in Table 1. 

A significant improvement can be obtained when initial conditions are taken into account in the FIFO service policy. In every Tx-slot, a counter is increased and when it reaches a preset threshold, a packet of the buffer is transmitted and the counter will be reset. This means that in FIFO, the packet is transmitted in that slot, while in Round-Robin, the new packet is stored in the buffer, waiting for its turn to be transmitted. In both cases, if the threshold is set to a different value, the offered traffic of the node will also be different.

At this point, it is convenient to define a set of variables for the Tx-slot counter *W*:

$W_{j,t}$ : Tx-slots elapsed for the node *j* (*j*=2,..,N) in the slot *t* since the last reset;
$W_{j}^{max}$ : preset threshold for $W_{j}$ (when $W_{j,t}=W_{j}^{max}$ a new packet is generated in slot *t* and $W_{j,t}=0$);
$W_{j}^{ini}$ : initial value for the counter of the node n ($W_{j,0}=W_{j}^{ini}$);
$W_{}^{ini}$ : vector containing $W_{j}^{ini}$ values for all nodes: $W_{}^{ini}=\left[\;W_{2}^{ini}\;\;W_{3}^{ini}\;\ldots \;\;W_{N}^{ini}\right]$.

The trivial case is to impose that $W_{}^{ini}=\left[0\;..\;0\right]$ when the network operation starts. However, testing for different values for every node, it is possible to obtain a lower average delay in the FIFO case, as can be seen in Table 2. In this table, two cases have been shown for the $W_{}^{ini}$ vector for three network sizes and different packet rates: the cases shown in Table 2 are the best and the worst average delay of all possible cases (permutations). For example, when using an initial vector of$\;W_{}^{ini}=\left[2\;4\;1\;0\;1\right]$ in the case of N=6 and uniform packet rate of λj= 110 , the maximum end-to-end delay is reduced to 13 slots, far from the case of 21 slots when using $W_{}^{ini}=\left[0\;1\;2\;4\;1\right]$. In this particular case, this is a reduction of 38%, a very important benefit for delay in the continuous operation of the string network proposed.

The number of packets delivered to the Gateway from every node is shown in the last column of Table 2. It can be seen that the two above mentioned $W_{}^{ini}$ vectors significantly reduce the delay with minimal impact on that number of packets. Similar results for the case of Round-Robin service policy can be observed in Table 3. 

**Figure 11:**
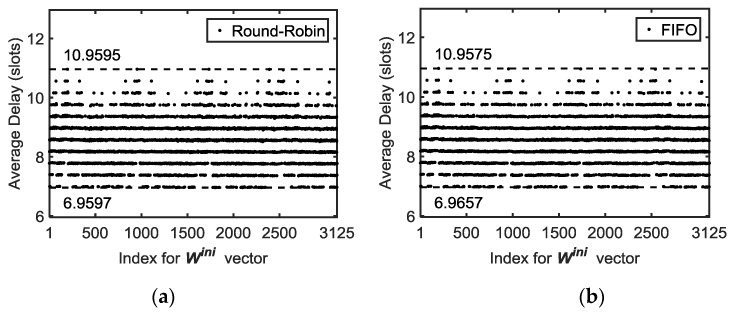
Average end-to-end delay sweeping $W_{}^{ini}$ values for an N= 6 nodes network and simulation time of 1000 slots. The two service policies considered (Round-Robin (**a**) and FIFO (**b**)) have similar values. The discretization in the *y*-axis is because the transmissions are allowed by the scheduler only at the beginning of a time slot.

Finally, if the maximum delay is the main design objective, Table 4 shows again a similar comparison between both policies, suggesting that the service policy hardly affects the end-to-end delay. This effect can be easily observed in Figure 11, where it can be seen how the service policy has a minimal effect on the bounds (min/max) of average end-to-end delay. In order to represent the simulation results for all the possible $W_{}^{ini}$ (permutations), an index vector has been used to name every different $W_{}^{ini}$ vector. This index is equal to the position of the vector $W_{}^{ini}$ in an ascending sorted list of all the permutations, i.e., index=1 for $W_{}^{ini}=\left[0\;0\;0\;0\;0\right]$, index=2 for $W_{}^{ini}=\left[0\;0\;0\;0\;1\right]$ and so on, up to index = 3125 (5^5^ different vectors) for $W_{}^{ini}=\left[4\;4\;4\;4\;4\right]$.

### 3.3. Queue Lengths

From the implementation point of view, it is very interesting to estimate the queue size needed to store packets in a node. The mean queue length $\bar{Q}$ is calculated as the overall sum of packets stored in the queues in all the time slots divided by the simulation time and the number of nodes (excluding the Gateway), that is:

(19) $$\bar{Q}=\frac{1}{\left(N-1\right)\cdot t_{sim}}\left(\sum_{j=2}^{N}\sum_{t=1}^{t_{sim}}r_{j,t}\right)\;,$$

with $r_{j,t}$ being the number of packets stored in the queue of node j after time slot t, and $t_{sim}$ the duration of the simulation. The results for both cases of Round-Robin and FIFO policies yield $\bar{Q}<1$ independently of N and the operation time $t_{sim}$ considered.

Regarding the maximum length of the queue, it is easy to see that there is an upper bound equal to the number of time slots that the node remains in a non-transmission state (i.e., receiving or idle) plus one (own packet generated). For the scheduling matrix $Q_{opt,4\times N}^{\left(4\right)}$ in Equation (11), the maximum queue length is three packets because the maximum time between transmission slots is 2.

### 3.4. Fairness

Throughput and fairness are usually in conflict. On one hand, the highest throughput is obtained with a greedy schedule. On the other hand, a fair schedule yields a poor throughput. When all node locations are equally important in terms of data acquisition, transmission fairness [28] is a scheduling objective. In this analysis, fairness means that all nodes transmit the same amount of their own data in the long-term, regardless of their distance to the sink node. Previous works by other authors deal with fair scheduling in STDMA networks. Wang et al. proposed a scheduling algorithm [29], but they emphasized adaptive scheduling instead of the shortest frame. Concerning UWSNs, Diamant and Lutz proposed STDMA protocol for ad hoc UWSNs where fairness was considered [30] but not uniformly achieved. Xiao et al. also presented an algorithm to find optimal fair scheduling for linear topology in TDMA networks [31], but the duration of the frame (called cycle in their paper) is greater than our frame length (four slots) and dependent of the network size N, i.e., for a N=3 network, their frame length is six slots, and 12 slots in the case of N=5. Besides that, the end-to-end delay is higher than the present case, as we have seen in Section 3.2. 

In general, a multi-hop TDMA network does not exhibit fair behavior. In our case, the optimal scheduling jointly with the packet generation rate in (14) guarantees a fair operation in the network, maintaining the number of packets delivered to the Gateway from every node. Moreover, the packet generation rate in (14) defines the maximum traffic load that a node can offer to the network, and fulfils the condition of queue stability:

(20) $$\sum_{j=2}^{N}\lambda_{j}\leq \frac{1}{2}$$

It is interesting to note that Equation (20) lets us adjust the traffic per node in the network to maintain a stable behavior in the long-term, and it is not imperative that if node *j* is farther than node ℓ from the Gateway, $\lambda_{j}\leq \lambda_{\ell }$ should happen to avoid bottlenecks.

In order to measure the differences between the average packet delays of the different nodes, a commonly used figure of merit of inequality is the Gini index [32]. Originally used in economics to show imbalances of income distribution [33], the Gini index has spread to many disciplines because of its simplicity, e.g., in demography (population studies) [34], medicinal chemistry [35], or even for improving the resources distribution in packet networks [36]. The Gini index G can be calculated from [37]:

(21) $$G\left(N\right)=\frac{1}{2\;N^{2}\;\bar{x}}\;\sum_{j=2}^{N}\;\sum_{\ell =2}^{N}\left|x_{j}-x_{\ell }\right|,$$

with N being the number of nodes, $x_{j}$ the average delay of the packets generated at node *j* and delivered to the Gateway, and $\bar{x}$ the arithmetic mean of $x_{j}$ (j=2..N). Index G takes values in [0,1]. Zero means a homogenous distribution or fairness, which means no difference, i.e., the average delays that the packets suffer from in terms of the different nodes would be the same. On the other hand, a value 1 for G means that there is a node with such a high average delay that the delays of the rest of nodes are negligible (usually called greedy behavior).

**Figure 12:**
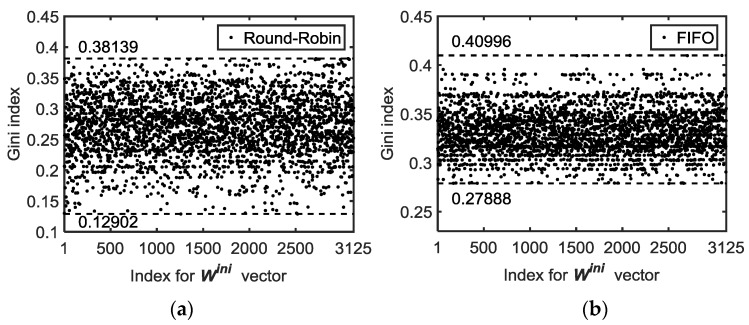
Gini index (*G*) for average end-to-end delay for two service policies: (**a**) Round-Robin and (**b**) FIFO. The bounds are shown in both graphs. Case data: *N*=6 nodes, simulation time: 1000 slots.

The simulation results for all possible $W_{}^{ini}$ (permutations) are presented in Figure 12 for a network with six nodes. In order to look for the bounds of the Gini index of the delay, Table 5 shows the minimum (fairest case) and maximum values for different network sizes under the same generated traffic conditions as those in Table 4.

When we observe Figure 12 and the results in Table 5, we realize that in, general, the Round Robin service policy is fairer, i.e., has a smaller Gini index. In all cases, however, a task-force analysis has to be carried out to find the best $W_{}^{ini}$ vector that achieves the sought objective, e.g., minimize the average delay, the maximum delay, or the Gini index.

## 4. Conclusions

This work addresses the scheduling and the service policy in relayed multi-hop underwater acoustic networks, so that maximum throughput, minimum delay, and network fairness are achieved. We propose a graphic procedure to determine the optimal case in a STDMA network with very long propagation delays. The network topology is linear and its purpose is sending packets from the network nodes to a sink node, or Gateway, located at one of the line edges. 

The method is based on a 2D space-time lattice and exploits the characteristic long propagation delay of the underwater acoustic channel and gives the optimal scheduling in terms of throughput. Analytic expressions are given to calculate the throughput and the results were verified by simulation. Two service policies, FIFO and Round Robin, have also been considered to analyze network delays and queue lengths. When fairness in terms of delay was taken into account, we observed that, in general, Round Robin is fairer than FIFO policy, while maintaining a similar number of packets that are delivered to the Gateway. An interesting point that came up while assessing the service policies is that fairness in terms of delay is sensitive to the initial condition of packet generation. 

## Figures and Tables

**Table 1:** Delay of the optimal scheduling (slots). Simulation time: 2000 time slots.

	Round-Robin		FIFO	
Size (N)	Max.	Average	Max.	Average
4	7	4	7	4
5	9	5.25	9	5.25
6	14	7.796	14	7.7965
7	17	8.996	17	8.996
8	19	11.9869	23	11.993
9	21	13.2329	25	13.2399
10	30	13.8843	28	13.8833
20	59	31.9368	63	31.9509
50 ^1^	193	81.8638	161	81.8637
100 ^2^	326	163.7764	327	163.7842

^1^ Simulation time: 5000 slots. ^2^ Simulation time: 10,000 slots.

**Table 2:** Delay of the optimal scheduling (slots) using FIFO. Simulation time: 1000 time slots.

Size (N)	Offered Traffic $\left[\lambda_{2}\;\lambda_{3}\ldots \;\lambda_{N}\right]$	$W_{}^{ini}$	Delay (Av./Max.)	Packets Delivered $\left[P_{2}\;P_{3}\ldots \;P_{N}\right]$
4	[ 18 18 16 ]	[2 1 0]	3.99/7	[125 124 166]
		[1 2 2]	5.29/11	[125 124 166]
4	[ 18 16 18 ]	[2 0 2]	3.69/7	[125 166 124]
		[1 1 3]	4.99/11	[125 166 124]
4	[ 16 18 18 ]	[2 1 0]	3.29/7	[167 124 124]
		[1 0 1]	4.69/11	[166 124 124]
5	[ 18 18 18 18 ]	[2 3 2 0]	4.98/9	[125 125 124 124]
		[3 0 1 3]	7.97/15	[125 124 124 123]
6	λj= 110 (j=2,..,6)	[2 4 1 0 1]	6.96/13	[100 100 99 99 98]
		[0 1 2 4 1]	10.95/21	[99 99 99 99 98]

**Table 3:** Delay of the optimal scheduling (slots) using Round-Robin. Simulation time: 1000 time slots.

Size (N)	Offered Traffic $\left[\lambda_{2}\;\lambda_{3}\ldots \;\lambda_{N}\right]$	$W_{}^{ini}$	Delay (Av./Max.)	Packets Delivered $\left[P_{2}\;P_{3}\ldots \;P_{N}\right]$
4	[ 18 18 16 ]	[2 1 0]	3.99/8	[125 124 165]
		[1 2 2]	5.29/11	[125 124 166]
4	[ 18 16 18 ]	[2 2 2]	3.69/7	[125 166 124]
		[1 0 3]	5/11	[124 165 124]
4	[ 16 18 18 ]	[2 1 0]	3.29/6	[167 124 124]
		[1 0 1]	4.69/8	[166 124 124]
5	[ 18 18 18 18 ]	[2 1 0 0]	4.98/10	[125 125 124 123]
		[3 0 1 3]	7.99/14	[124 124 124 124]
6	λj= 110 (j=2,..,6)	[2 4 2 0 0]	6.96/13	[100 100 100 98 98]
		[1 2 3 0 2]	10.96/16	[99 99 99 99 98]

**Table 4:** Maximum delay of the optimal scheduling (slots) Round-Robin vs. FIFO. Simulation time: 1000 time slots.

		FIFO		Round-Robin	
Size (N)	Offered Traffic $\left[\lambda_{2}\;\lambda_{3}\ldots \;\lambda_{N}\right]$	$W_{}^{ini}$	Delay Max. (Av.)	$W_{}^{ini}$	Delay Max. (Av.)
4	[ 18 18 16 ]	[1 2 2]	11 (5.29)	[1 2 2]	11 (5.29)
4	[ 18 16 18 ]	[1 1 3]	11 (5)	[1 0 3]	11 (5)
4	[ 16 18 18 ]	[1 0 1]	11 (4.69)	[0 2 3]	11 (4.69)
5	[ 18 18 18 18 ]	[3 0 1 3]	15 (7.99)	[3 0 1 3]	14 (7.99)
6	λj= 110 (j=2,..,6)	[0 1 2 4 1]	21 (10.95)	[0 1 1 4 0]	21 (10.16)

**Table 5:** Bounds for delay Gini index (optimal scheduling). Simulation time: 1000 time slots.

		FIFO		Round-Robin	
Size (N)	Offered Traffic $\left[\lambda_{2}\;\lambda_{3}\ldots \;\lambda_{N}\right]$	Gini Min. [$W_{}^{ini}$]	Gini Max. [$W_{}^{ini}$]	Gini Min. [$W_{}^{ini}$]	Gini Max. [$W_{}^{ini}$]
4	[ 18 18 16 ]	0.28540 [3 2 0]	0.37605 [2 1 1]	0.19829 [2 3 1]	0.37592 [3 2 2]
4	[ 18 16 18 ]	0.30671 [2 2 1]	0.35603 [0 1 3]	0.19432 [2 1 1]	0.36545 [1 0 1]
4	[ 16 18 18 ]	0.26642 [0 1 2]	0.39152 [2 2 3]	0.20587 [0 1 0]	0.3314 [2 3 3]
5	[ 18 18 18 18 ]	0,25064 [1 1 1 1]	0.41686 [3 0 1 1]	0.13415 [3 0 2 2]	0.40385 [1 0 2 1]
6	λj= 110 (j=2,..,6)	0.27888 [4 0 0 0 3]	0.40996 [3 1 3 1 2]	0.12902 [2 0 0 1 3]	0.38139 [4 4 2 3 1]
7	λj= 112 (j=2,..,7)	0.30 [0 5 3 0 4 3]	0.42658 [4 2 1 0 4 0]	0.20 [0 4 4 4 4 2]	0.40805 [5 5 1 4 4 0]
